# Graphene Oxide-Carbon Nanotube (GO-CNT) Hybrid Mixed Matrix Membrane for Pervaporative Dehydration of Ethanol

**DOI:** 10.3390/membranes12121227

**Published:** 2022-12-05

**Authors:** Oindrila Gupta, Sagar Roy, Lingfen Rao, Somenath Mitra

**Affiliations:** Department of Chemistry and Environmental Science, New Jersey Institute of Technology, Newark, NJ 07102, USA

**Keywords:** pervaporation, ethanol dehydration, mixed matrix membrane, carbon nanotube, graphene oxide

## Abstract

The pervaporation process is an energy-conservative and environmentally sustainable way for dehydration studies. It efficiently separates close boiling point and azeotrope mixtures unlike the distillation process. The separation of ethanol and water is challenging as ethanol and water form an azeotrope at 95.6 wt.% of ethanol. In the last few decades, various polymers have been used as candidates in membrane preparation for pervaporation (PV) application, which are currently used in the preparation of mixed matrix membranes (MMMs) for ethanol recovery and ethanol dehydration but have not been able to achieve an enhanced performance both in terms of flux and selectivity. Composite membranes comprising of poly (vinyl alcohol) (PVA) incorporated with carboxylated carbon nanotubes (CNT-COOH), graphene oxide (GO) and GO-CNT-COOH mixtures were fabricated for the dehydration of ethanol by pervaporation (PV). The membranes were characterized with Scanning Electron Microscopy (SEM), Fourier Transform Infrared Spectroscopy (FTIR), Thermogravimetric Analysis (TGA), Differential Scanning Calorimetry (DSC), Raman spectroscopy, Raman imaging, contact angle measurement, and water sorption to determine the effects of various nanocarbons on the intermolecular interactions, surface hydrophilicity, and degrees of swelling. The effects of feed water concentration and temperature on the dehydration performance were investigated. The incorporation of nanocarbons led to an increase in the permeation flux and separation factor. At a feed water concentration of 10 wt.%, a permeation flux of 0.87 kg/m^2^.h and a separation factor of 523 were achieved at 23 °C using a PVA-GO-CNT-COOH hybrid membrane.

## 1. Introduction

Among different biofuels in the market today, bio-ethanol is the most common and is extensively used as a gasoline additive [1,2]. Efficient purification techniques are needed to obtain high purified ethanol from fermentation processes [3], which in fact accounts for 60–80% in the total production cost. Conventional separation techniques include thermal distillation [4], low temperature crystallization, adsorption [5,6], and extraction [7,8]. Distillation is the most commonly used process [9], however typical distillation can only produce ethanol with a purity close to 95% (by wt.) owing to the formation of water-ethanol azeotrope. Water and ethanol mixture (4.37 wt.% water) forms an azeotrope at 78.2 °C. Thereby, it is a difficult task to produce pure ethanol from an azeotropic mixture by conventional distillation: at the azeotrope vapor and liquid compositions are the same. The azeotropic distillation involves the addition of an “entrainer” to the distillation process which is not only energy-consuming, but also environmentally unfriendly [10]. Herein, the PV has been introduced as a promising alternative towards such purpose.

Membrane-based separations, specifically pervaporation (PV) which is a combination of membrane permeation and evaporation has evolved to be a promising method for ethanol dehydration. It has several advantages over conventional methods including the separation of constant-boiling azeotropes and heat sensitive products with minimal energy consumption owing to its low operating temperature and pressure. It has also been used with a variety of solvents [11,12,13,14]. The key to the success of PV is the fabrication of appropriate membranes with high permeability and selectivity [15]. Physical and chemical modifications such as blending of polymers [16,17] and the adding of solid filler has been used to improve solute-membrane interaction via preferential adsorption, resulting in an increase in separation factor and flux [18,19]. One of the most successful trends in improving the performance of polymeric membranes applies embedding of inorganic materials, generating the so-called mixed matrix membranes (MMMs). These combine the strengths of inorganic and polymeric membranes to ideally reach an enhanced synergistic performance. Mixed matrix membranes (MMMs) consisting of inorganic moieties embedded inside a polymer matrix, have also been developed for PV [20]. Polyvinyl alcohol (PVA) is the most popular PV membrane owing to its high hydrophilicity, superior process ability, and presence of hydroxyl groups for easy post modification [21,22]. However, PVA-based membranes have poor mechanical strength and stability in the aqueous solution due to the excessive swelling phenomenon, resulting in decline in the separation performance. Crosslinking modification [23,24,25,26] have been used as an effective method to develop stable and robust membranes, and numerous crosslinking reagents, including glutaraldehyde [27,28], amic acid [13], and fumaric acid [29], have been investigated. An alternative approach to modify PVA has been to incorporate with inorganic moieties [30], including zeolite [31], silica [32], activated carbon [33], carbon nanotube [34,35], metal oxide [36]. An enhanced separation performance using the appropriate filler has been demonstrated, however, such developments depend upon compatibility between nanoparticle and polymeric phases [37].

Nanofillers are widely used in membrane separation processes to achieve enhancement in performance over conventional membranes available in the market for various applications, ranging from membrane distillation to pervaporation [38,39,40,41,42,43]. Among the various fillers investigate for mixed matrix membranes [33,44,45], carbon nanotubes (CNTs) have received significant attention as an additive in PV membranes and have shown improvement in performance [34]. The as-produced CNTs tend to agglomerate into bundles due to strong van der Waals forces [46], consequently attaching functional groups to the CNTs is an efficient method to avert aggregation, which aids in better dispersal of the CNTs in a polymer matrix [47]. Similarly, graphene oxide (GO) based composites which utilize the numerous oxygen-containing functional groups (e.g., hydroxyl, epoxide, and carboxyl groups) have shown enhancement when incorporated in PVA membranes during organic-organic separations [48,49]. In short, both CNTs [39,42,50] and GO [51,52,53,54] are promising material for membrane fabrication and can have potential benefits for the development of PV membranes.

Recently, GO had been shown to disperse CNTs in an aqueous matrix to form an integrated three-dimensional network through π-π interaction [55]. The combination of one-dimensional CNTs and two-dimensional GO nanosheets lead to the formation of a layered structure where the CNTs serve as spacers between the GO sheet, which prevents the GO sheets from collapsing onto each other. This forms nanovoids that serves a network for solute permeation. The combination of CNTs and GO have shown to act as great reinforcing fillers in polymer nanocomposites [56] due to their excellent physical properties, as a versatile glucose biosensor [57] and used as a transparent conductor in optoelectronic devices [58]. Previous studies have shown that membrane permeation through GO sheets occurs via nanocapillary effects, while the permeation on CNTs is based on sorption and rapid mass transfer [38,40]. A comparison table has been shown in Table 1 to summarize already published work. The synergistic effect of these two nanocarbons in a GO-CNT hybrid format is yet to be explored in membrane separations. The objective of this paper is to synthesize PVA based GO-CNT hybrid membranes for azeotropic separation of ethanol-water mixtures.

## 2. Experimental

### 2.1. Materials

99%+ hydrolyzed PVA with high molecular weight of 146,000 was used (Sigma-Aldrich, St. Louis, MO, USA). Ethanol (200 proof) was obtained from Sigma Aldrich (St. Louis, MO, USA). Glutaraldehyde (25% concentration in water, Sigma Aldrich, St. Louis, MO, USA), hydrochloric acid (0.1N, extra pure grade), CNTs (Cheap tubes Inc., Cambridgeport, VT, USA), Graphene Oxide (Fluka, Everett, DC, USA) were extra pure grade. The functionalized CNTs (CNT-COOH) were prepared by bonding the carboxyl functional group on the multiwalled CNT sidewall through microwave-induced reaction in a Microwave Accelerated Reaction System (CEM Mars, Model Number 907501, Spectra Lab Scientific Inc., Alexandria, VA, USA), as stated in our previous paper [67]. The water used was deionized water for preparing the aqueous feed solutions for the pervaporation experiments. All chemicals were used without any further purification.

### 2.2. Membrane Preparation

An appropriate casting method was used to prepare the membrane. The technique of membrane preparation follows a procedure reported in references [68,69,70]. Membrane film casting followed by solvent evaporation and drying were performed according to literature [71]. Water was used as the dissolving solvent for PVA by stirring for 6 h at 80 °C. A clear well mixed solution of 3 wt.% PVA in water was obtained. To this solution, hydrochloric acid (HCl, 0.1 N), which acted as a catalyst and a predetermined quantity of crosslinking agent (25% solution, in the ratio of 1:0.05 (PVA: crosslinking agent), glutaraldehyde (GA) was added. The solution was slowly stirred continuously at room temperature for a desired amount of time (60 min) and was then kept overnight for bubbles to disappear. Then, the cooled and bubble-free solution was cast on a glass plate by a casting knife. Casting knife gap was adjusted after several trial and error in the membrane fabrication process. The final thickness of the membrane after drying was found ~55 micron. Finally, the membranes were cross-linked in a vacuum oven at temperature 60 °C for 8 h. For PVA-GO and PVA-CNT-COOH composite membrane, 0.1 wt.% of nanomaterials were added in PVA solution.

### 2.3. Membrane Characterization

Scanning electron microscopy (SEM, model JSM-7900F) was used for the investigation of the PVA membrane morphology. The crosslinking reaction of PVA with GA was confirmed by the Fourier transform infrared spectroscopy (FTIR) with a FTIR spectrometer (Agilent Cary 600 Series). Thermo-oxidative stability of the polymer films was examined, using thermogravimetric analysis (Perkin Elmer TGA 8000), from 25 to 600 °C heated at 10 °C/min. Raman spectra and Raman images were recorded using Thermo DXR2xi Raman Imaging Microscope with a laser excitation wavelength 532 nm, grating 900 lines/mm and spectrum range 3400–50 cm^−1^.

### 2.4. Sorption Studies

Weighed samples of cross-linked polymer films were soaked in aqueous ethanol (5, 10 and 20 wt.% water) solution; however, for brevity, sorption data for 90 wt.% ethanol in water has been reported here. Similar trend was observed for the other conditions tested. The films were taken out after different soaking periods and quickly weighed after carefully wiping out excess liquid from the surface to determine the amount of solution absorbed periodically and quickly positioned back in the solvent. The process was continued until the films attained a steady state as indicated by constant weight. The degree of swelling was calculated from the equation [72]:

(1) $$Degree\;of\;swelling=\frac{W_{s}}{W_{D}}\;\;$$

where *W_s_* is the weight of the swollen polymer in grams and *W_D_* is the weight of the dry polymer in grams.

The percent sorption is obtained from Equation (2)

(2) $$\%\;Sorption=\frac{W_{s}-W_{D}}{W_{D}}*100$$

## 3. Pervaporation Experiments

**Figure 1:**
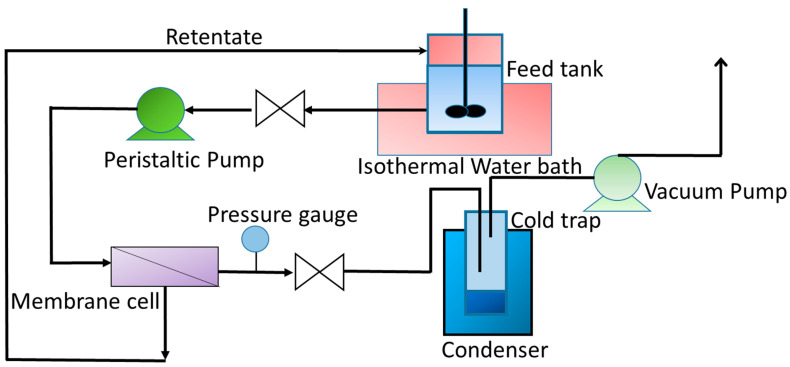
Schematic of PV experimental setup.

The PV experiments were performed as shown in the schematic of the experimental set-up presented in Figure 1. The initial feed volume was 200 mL. The feed was circulated at atmospheric pressure. However, a slight increment in pressure ~2–3 psig has been observed in the feed side. A very small amount of sample (~1 mL) was needed for the accurate analysis of the permeate composition using a refractometer. The aqueous ethanol feed mixture at varied concentrations were circulated through the pervaporation cell from a feed tank using a pump with a constant flowrate of 30 mL/min. The pressure at the downstream side was kept about 6 mbar using a vacuum pump. Permeate was condensed using traps immersed into liquid nitrogen. Pervaporation flux was determined by weighing the amount collected by the liquid nitrogen trap during a given time after equilibrium reached for all experiments. The feed solution temperature was varied in the range of 23–50 °C and the area of the membrane in contact with the feed solution was 14.5 cm^2^. The composition of the feed mixture was analyzed by measuring the refractive index of the permeate by a high-precision Refractometer (EW 81150-55, Cole Parmer) with an accuracy of ±0.0001 units and then plotting a calibration curve of refractive index versus known compositions of ethanol water mixtures to determine the unknown composition of the vapor penetrant collected mixture. The permeation flux (*J*_w_) was calculated from the following equation [72]:

(3) $$J_{w}\left(\frac{g}{m^{2}}\cdot \;h\right)=\frac{W\;\left(g\right)}{A\;\left(m^{2}\right)*t\left(h\right)}$$

where *W* is the weight of permeant, *A* and *t* is the effective membrane area (*m*^2^) and experimental time, respectively.

The water separation factor (*α*_*water*_) was calculated by:

(4) αwater=ywateryethanolxwaterxethanol

where *x_water_*, *x_ethanol_* and *y_water_*, *y_ethanol_* are the weight fractions of water and ethanol in the feed and permeate, respectively.

## 4. Results and Discussion

### 4.1. Characterization of the Crosslinked PVA Membranes

**Figure 2:**
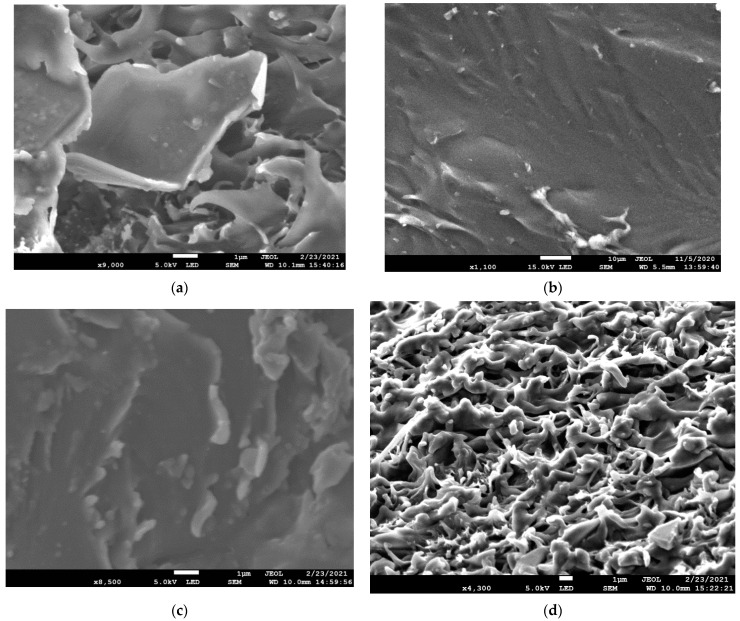
Cross sectional SEM images of (**a**) PVA; (**b**) PVA-GO; (**c**) PVA-CNT-COOH; (**d**) PVA-GO-CNT-COOH nanocomposite membranes.

Scanning electron micrographs of the crosslinked PVA membranes are presented in Figure 2a–d. Figure 2a shows the cross-section of the crosslinked PVA membranes. The membrane sample for cross-section SEM analysis was prepared by fracturing the membrane using liquid nitrogen. The cross-sectional morphologies of the composite membranes are presented in Figure 2b–d, where the CNT-COOH content was 0.1 wt.% of the polymer. The CNT-COOH were well dispersed within PVA matrix, which indicated that functional groups are a suitable way to alleviate the serious aggregation of CNTs. A well-distributed and tightly packed distribution of GO can be seen in nanocomposites. GO platelets more than 7 mm in length and about 50–60 nm thickness can be seen. However, it appears that the cavities between the laminates are marginally greater in PVA-GO. A uniform distribution of GO and CNT-COOH is seen in the hybrid membrane cross-sectional SEM images.

#### 4.1.1. FTIR Analysis

**Figure 3:**
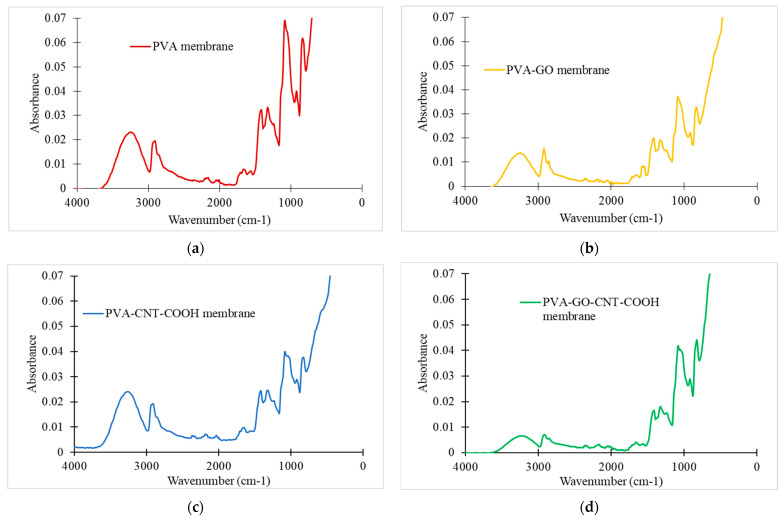
FTIR Analysis of (**a**) PVA; (**b**) PVA_GO; (**c**) PVA_CNT_COOH and (**d**) PVA_GO_CNT_COOH membranes.

Yeom and Lee [73] have investigated crosslinking reaction between PVA and GA and HCl that acted as a catalyst. Figure 3a shows the infrared absorption spectra of the PVA polymer with GA crosslinking. The characteristic absorption peaks are: one discrete wide absorption bands at 3000–3700 cm^−1^, which is accredited to widening of the -OH hydroxyl group; the peak at 2900 cm^−1^ conforming to irregular and symmetrical stretching of the C-H molecule; 1710 cm^−1^, equivalent to stretching of C=O group of aldehyde; 1650 cm^−1^, stretching of C-C group, 1210–1470 cm^−1^, could be attributed to variable deformation vibrations of the CH_2_ or CH groups; 1094 and 1200 cm^−1^, which are attributed to C=O and C-O-C groups due to acetal or ether development. The PVA–GO, PVA-CNT-COOH and PVA-GO-CNT-COOH nanohybrid membranes were characterized by FTIR spectroscopy. As shown in Figure 3b–d, the peak at 3200 cm^−1^ attributed to the –OH group sharply increased. These changes suggest that the surface of the polymer substrate was sufficiently covered by the PVA–GO nanohybrid sheets.

The chemical structures of CNT-COOH were studied by FTIR. C=O stretching vibration of the carboxylic groups can be observed with a peak at 1690 cm^−1^. The FTIR spectra of PVA blend films show the characteristic bands at 1070 cm^−1^ (–C–O stretching), 1300 cm^−1^ (crystallization-sensitive band of PVA) and 1590 cm^−1^ (–NH_2_ bending). The hydrogen-bonded hydroxyl group of the PVA polymer can be seen at 3000–3500 cm^−1^.

The MWCNTs without functional groups, in theory, should cause no shift in chemical bonding to the PVA matrix when integrated into the polymer matrix. The MWCNTs primarily interacted with the neighboring PVA polymer matrix by interfacial linkage, which comprised of physical interfaces and mechanical interlinking [74]. In contrast, the –COOH groups that were covalently attached to the carbon atoms could result in hydrogen bonded clusters with the OH groups from the base polymer [75].

#### 4.1.2. TGA Studies

**Figure 4:**
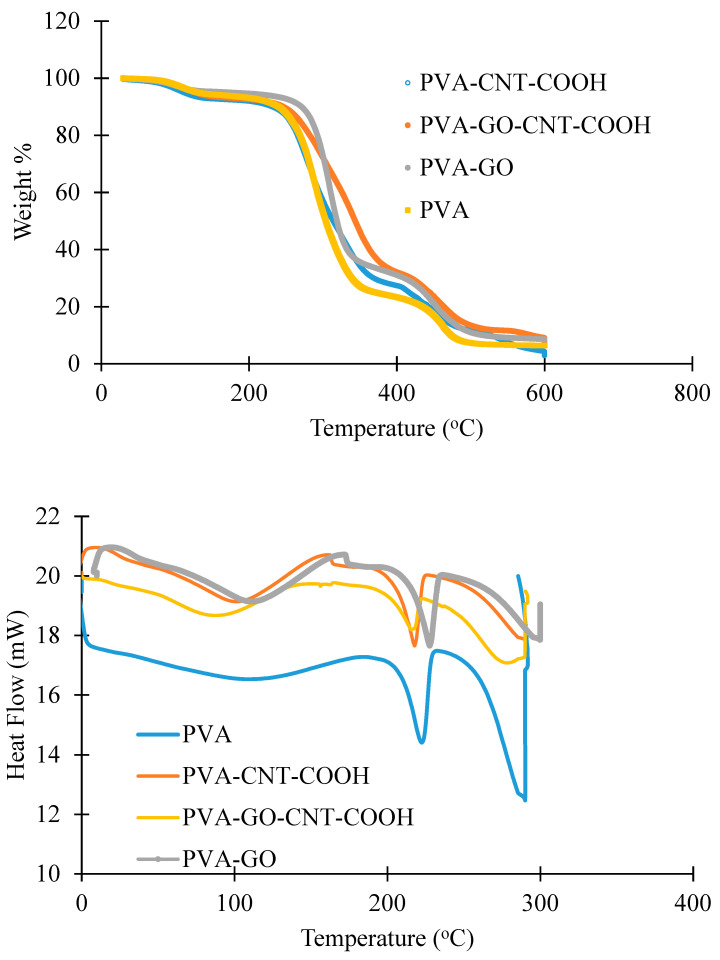
TGA and DSC curves of PVA and nanomaterial based PVA membranes.

Figure 4 shows the TGA results for pure PVA membrane and the fabricated membranes. The TGA curve of pristine PVA membranes shows three main degradation stages followed by the final decomposition of the polymer blend. The presence of water inside the PVA matrix partially exists as absorbed water molecules (weak bonds) on the exterior or interior surface without reacting with the matrix, and to some extent present as water molecules that are firmly attached to the hydroxyl groups. The first weight loss region in the TGA curve at around 100 °C can be attributed to the loss/evaporation of the absorbed water, while the next one between 250–370 °C is associated to the deficiency of water attached to the polymer matrix and polymer degradation. The third region between 400–600 °C is associated with the disintegration and carbonization of the polymer [76]. According to the TGA curves, it was obvious that the temperatures of the weight reduction areas increasingly improved with the existence of nanomaterials in the PVA matrix, indicating improved stability of the PVA/GO/CNT-COOH membranes at elevated temperatures.

DSC analysis is used for the prediction of amount of heat taken up or released when materials endure physical or chemical alterations. The initial heating scan stage is typically performed to disregard any heat related history of a sample and for moisture content decline. Consequently, the subsequent heating phase was considered in this DSC study. Figure 4 shows DSC curves for the pure PVA and PVA- nanomaterial composites. The glass transition temperature, Tg value of pristine PVA membrane was 65 °C, and it increased to 83 and 102 °C for the PVA-CNT and PVA-GO-CNT-COOH membranes, respectively. As can be seen, all the nanofibers exhibited an heat-absorbing peak around 210–235 °C, equivalent to the melting temperature of PVA.

#### 4.1.3. Raman Spectra and Raman Imaging of Fabricated Membranes

**Figure 5:**
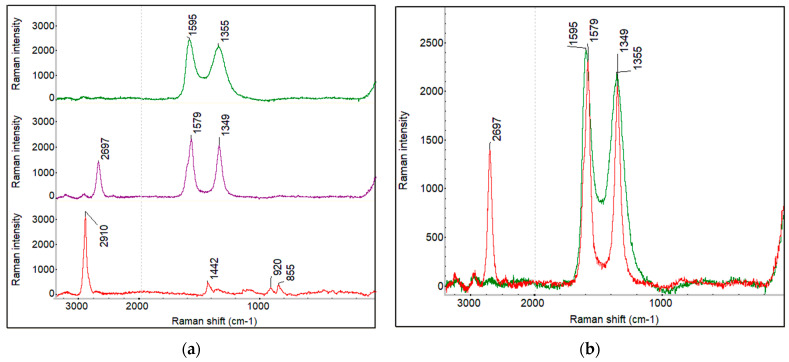
(**a**) Raman spectra. From top to bottom: GO powder, CNT_COOH powder, and PVA film. (**b**) Raman spectra overlay of GO powder (green) and CNT_COOH powder (red).

Yang et al. [77] showed the micro-Raman spectra of PVA powders and the PVA polymer membrane, which shows some strong characteristic scattering peaks of the PVA polymer at 1440, 1145, 926, 852 cm^−1^, respectively. Raman spectra of GO powder exhibit a strong D-band (~1360 cm^−1^) and G-band (~1590 cm^−1^) usually assigned to structural disorder and the graphitized structure, respectively, as shown in the literature [78]. GO presents a dominant D peak arising from the attachment of functional groups such as hydroxyl and epoxy on the carbon skeleton. The D + D′ peak (~2950 cm^−1^) represents a defect activated combination of phonons with different moments. The large D-band and the attenuated 2D-band may be attributed to the small sp^2^ domains subsequent to the oxidation process and ultra-sonication during synthesis. Figure 5a displays the representative Raman spectra of PVA film, GO powder, and CNT-COOH powder. Figure 5b shows the Raman spectra overlay of GO powder (green) and CNT-COOH powder (red). The GO and CNT-COOH have two common bands D and G and differ in 2D band. G band around 1575–1600 cm^−1^ of the in-plane vibration of the six-membered ring common to carbon-based substances are observed in both CNT–COOH and GO. D-band associated with defects in the carbon structure is observed at 1345–1360 cm^−1^ in both GO and CNT-COOH. Both the D and G bands are sharper in CNT-COOH than GO. The Raman intensity ratio of G band/D band in CNT-COOH is large compared to CNT reported in the literature, which is consistent with the modification of the carbon nanotubes. 2D band is present in the CNT-COOH at 2697 cm^−1^, while absent in GO band. This difference allows mapping the CNT distribution in the PVA-GO-CNT film using the 2D band. The Raman spectrum of the PVA film has a strong peak at 2910 cm^−1^. This is the characteristic C-H stretching band of the organic polymer. The C-H stretching band is well separated from the 2D band of the CNT-COOH and this band is used to map PVA distribution in the composite membranes PVA-CNT-COOH and PVA-GO-CNT-COOH.

##### Optical and Raman Imaging of PVA Membrane

The Raman Microscope provided high resolution optical images which was complemented by the detailed chemical image generated by Raman spectroscopy. Correlation mapping and multivariate curve resolution MCR analysis was combined to map the components in the sample. In this study, the spatial distribution of the nanomaterials on top of the membrane surface was studied. Raman microscopy helped analyze selected sample area with a high spatial resolution. For brevity, Raman imaging of plain PVA membrane and hybrid PVA-GO-CNT-COOH membrane is discussed here.

**Figure 6:**
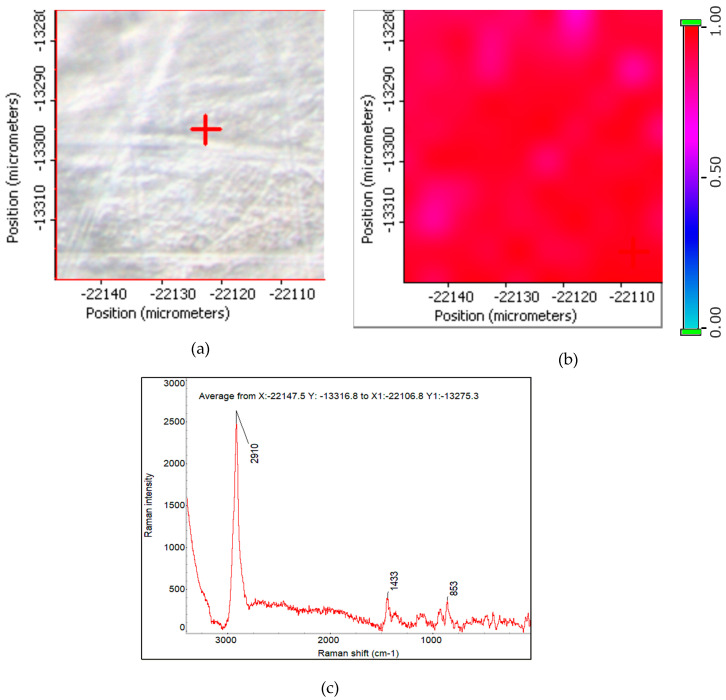
(**a**): Raman image of PVA membrane; (**b**) Optical image of the same area 70 um × 70 um. (**c**) The average spectrum of the Raman mapping area (it is used as correlation spectrum).

A representative area of 450 × 300 um of the PVA film was surveyed with Raman spectra. The Raman spectral survey observed only PVA as expected. A region of 70 um × 70 um was selected for Raman imaging. Figure 6a,b are the Raman correlation image (profile) and the optical image of the same area. The correlation image shows the correlation between each sample point in the Raman chemical image and the reference PVA spectrum shown in Figure 6c. Spectra that are exactly like the correlation reference have a correlation value of 1.00 on the profile intensity scale and are displayed in the color at the top of the scale. The intense red color across the imaged area shows uniform distribution of PVA.

##### Raman Imaging of PVA_GO_CNT_COOH

**Figure 7:**
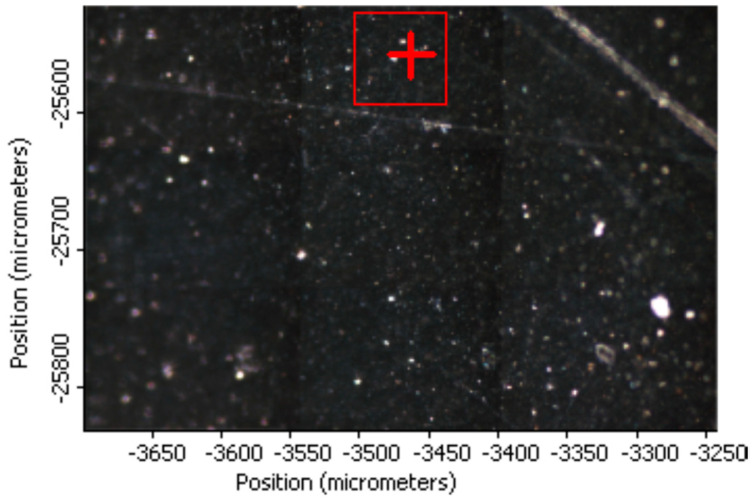
Optical image of PVA_GO_CNT_COOH sample with a representative area of 450 um × 300 um.

Figure 7 is the optical image of PVA-GO-CNT-COOH sample with a representative area of 450 um × 300 um. The PVA-GO-CNT-COOH film appears uniformly black in the optical image. The blurry and bright spots on the black membrane seen in Figure 7 are surface contamination and are due to dusts on the membrane surface.

**Figure 8:**
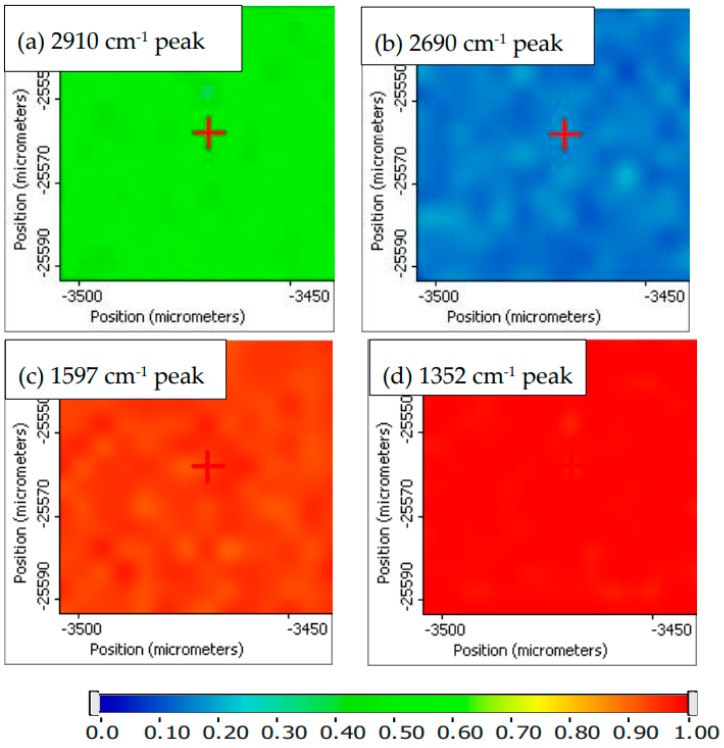
Raman images obtained using peak height profiling option of normalized Raman spectra for the PVA_GO_CNT_COOH membrane in a region of 65 um × 65 um. These images are the peak height distribution for (**a**) PVA, (**b**) CNT_COOH, (**c**) GO_CNT-COOH, and (**d**) GO_CNT_COOH in the composite hybrid membrane.

**Figure 9:**
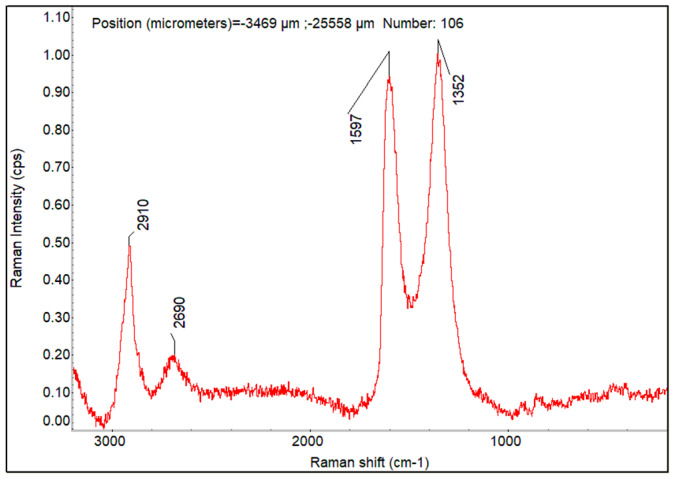
A representative Raman spectrum of the hybrid membrane extracted from the Raman imaged area at the cross-hair location in Figure 8.

The Raman spectral survey of the 450 um × 300 um area suggests the distribution of PVA, GO, and CNT-COOH is quite uniform. A small region of 65 um × 65 um marked red in Figure 7 was selected for Raman imaging. The Raman peak intensities at 2910, 2692, 1597, and 1352 cm^−1^ were used to map the distribution of PVA, CNT, and GO + CNT-COOH in the composite hybrid membrane. The obtained Raman images using peak height profiling option of normalized Raman spectra for the PVA-GO-CNT-COOH membrane are shown in Figure 8a–d. A representative Raman spectrum of the hybrid membrane extracted from the Raman imaged area at the crosshair location in Figure 8 is shown in Figure 9. The Raman imaging results suggest that GO and CNT-COOH distribute quite evenly within PVA. No local concentration of either component was detected by Raman imaging.

Further, Raman correlation image was calculated for the same Raman data set shown in Figure 8. The obtained Raman correlation image showed the uniform red color, clearly indicating the hybrid membrane has a uniform surface distribution of the three chemical components.

#### 4.1.4. Contact Angle Measurements

**Figure 10:**
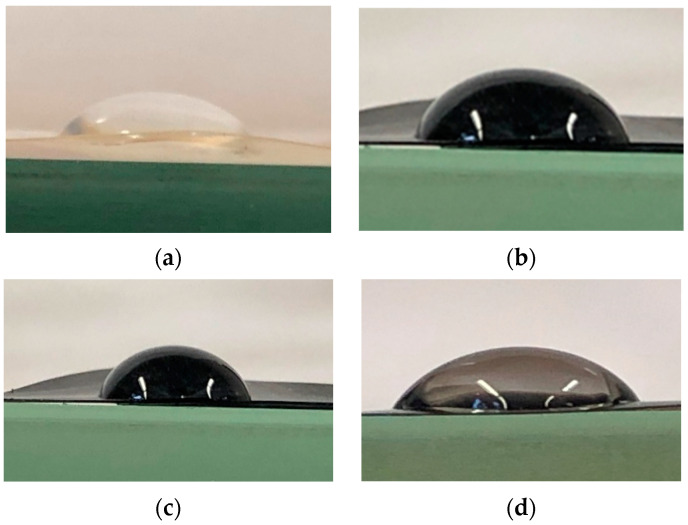
Contact angle of water in the presence of (**a**) PVA film; (**b**) PVA_CNT_COOH; (**c**) PVA_GO_CNT_COOH; (**d**) PVA_GO.

Figure 10 shows the contact angle images of water on PVA film, PVA_CNT_COOH, PVA_GO_CNT_COOH and PVA_GO membranes. The contact angle measurements were performed to determine affinity. Table 2 summarizes the contact angles obtained for pristine PVA and fabricated membranes. The measured water contact angle value for pristine PVA membrane was around 69° which is in agreement with data published by other researchers [79]. On the other hand, the PVA membrane exhibited an enhanced hydrophilicity by embedding GO into its matrix as shown in Figure 10d. The contact angle for PVA-GO was estimated to be 65° ± 0.5°, showing the presence of hydrophilic functionalities on the surface of GO. The water contact angle increased to 75° in the case of PVA-CNT-COOH due to a decrease in hydrophilicity in the presence of hydrophobic CNTs. The contact angle measurement for GO/CNT-COOH hybrid on PVA membrane is 71°. The wetting behavior between liquid and solid is favored by high solid surface energy [80]. Previous research had shown a decrease in hydrophilicity with GO in other mixed matrix membranes based on chitosan [81] and polyimides [82]. In principle, membrane wettability has a direct relationship with the rate of water adsorption on the surface of the membrane. Water sorption is a primary step in pervaporation based on the on the solution-diffusion mass transfer mechanism and thus contact angle measurements provide a clear idea on the transport properties through the membrane.

## 5. Pervaporation Experiments Results

### 5.1. Pervaporation

**Figure 11:**
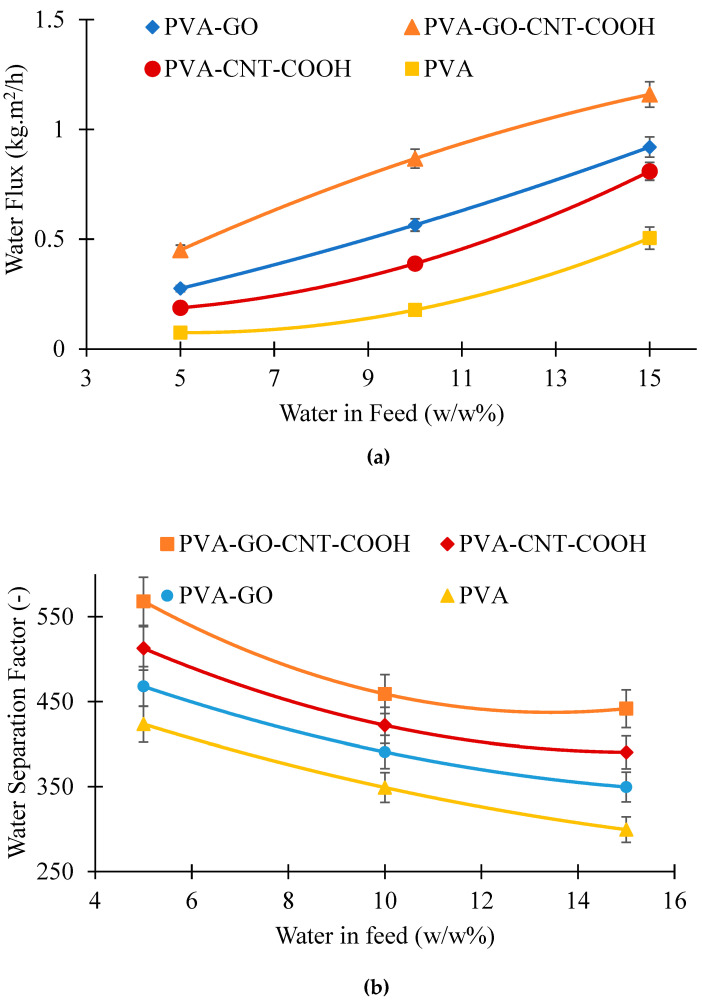
Effect of feed concentration on (**a**) water flux; (**b**) water separation factor at 50 °C.

Figure 11a shows the performance of the membranes in terms of flux and separation factor for separating water–ethanol mixtures with varying water concentration in the feed mixture. All membranes investigated were able to selectively permeate water. The permeate flux of water reached as high as 1.92 kg/m^2^.h at 15 wt.% water and 50 °C temperature for PVA-GO-CNT membrane showing an enhancement of 32% over pristine PVA membrane. PVA-CNT-COOH had a lower permeate flux than PVA-GO membrane. It is believed that the addition of CNTs into the membrane matrix reduces the free spaces as the CNTs act as reinforcing bridge elements, increases rigidity of the polymeric chains and thus the permeation flux decreases [83]. At 10 wt.% water in the feed and 40 °C, 86 wt.% water was obtained in the permeate with the chemically crosslinked pristine PVA membrane, and the PVA-GO-CNT-COOH membrane yielded a better separation factor. This is shown in Figure 11b, where the separation factor for the different membranes is mapped against feed water concentration. An assessment of the selectivity of membranes show that the CNT-GO hybrid membranes were improved compared to membranes made from either PVA-GO or pristine PVA, particularly in the range of 5–15 wt.% water in feed. PVA-GO-CNTCOOH membrane showed an enhancement of 24% over unmodified PVA membrane at room temperature and 10 wt.% water in the feed.

As seen in Figure 11b, the separation factor (α) for all the membranes decreased with an increase in water concentration in the ethanol–water solution with the highest selectivity obtained for PVA-GO-CNT-COOH membranes. The flux for both water and ethanol increased with an increase in the water concentration in the feed solution. As reported by Bartels [84], the attributes of the sorption were based on the variation among the likeness of the elements to the polymer, the shared interactions of the element, and the pattern in which the interactions with the polymer of each element impacts the interactions of the additional penetrates with the polymer. Commonly, hydrophilic membranes have superior affinity towards water than ethanol, and the smaller water molecules make diffuse faster than ethanol. However, due to the intense interaction between the water and ethanol, the separation factor decreases with an increase in flux. Besides, the α of the PVA-GO-CNT-COOH composite membrane was higher than that of the PVA-GO and PVA-CNTCOOH membranes. An increase in feed water concentration not only represented an increase in the driving force, but also enhanced the membrane swelling, thus enhancing the diffusion of the permeant molecules.

#### Effect of Ethanol–Water Solution Temperature on Pervaporation Performance of PVA, PVA_GO, PVA_CNT_COOH and PVA_GO_CNT_COOH Membranes

**Figure 12:**
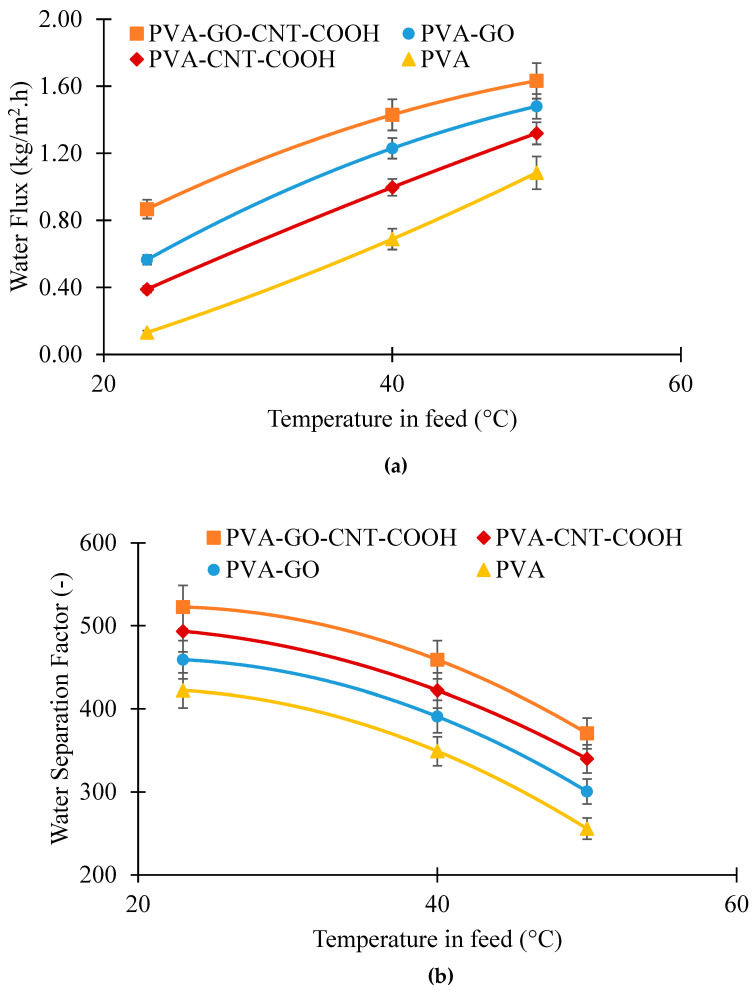
Effect of feed temperature on (**a**) water flux; (**b**) water separation factor at 10 wt.% water in feed.

As can be seen from Figure 12a, the total permeation increased with feed temperature for all fabricated membranes at 90 wt.% feed ethanol. This was attributed to the fact that the increase in feed temperature elevated the diffusion coefficient and led to an increase in bulk mass transfer through the membrane [85]. In addition, the increase in feed temperature could result in structural changes in the membrane that results in prior phase shift of liquid within the membrane as the necessary heat content for the shift extends quicker when there is more heat provided. According to Figure 12b, separation factor for all membranes decreased with the increase in feed temperature, however, the decline was significantly less in the presence of nanomaterials incorporated into the pristine PVA membrane. It implies that amount of water in permeate with the presence of GO and CNT-COOHs tended to be constant or had a slight decline while that of pristine PVA membrane inclined to reduce with the feed temperature. This reduction in water amounts in permeate upon increasing the feed temperature could be because the expansion of thermal flexibility of polymer chains lets both water and ethanol transfer through membrane, ultimately reducing the separation factor. Separation factor reached as high as 522 and 493 with PVA-GO-CNT-COOH and PVA-CNT-COOH, respectively, showing an enhancement of 32% and 21% over pristine PVA membrane at 40 °C. These experimental findings are in agreement with other researchers [86,87].

When the temperature is raised, the saturated vapor pressures of water and ethanol are enhanced, resulting in higher driving force for mass transport across the membrane. Additionally, according to the solution-diffusion model, the permeability of membranes is governed by the diffusivity and solubility of the permeant. With higher temperature, the permeant develops more energy with a rise in motion of the polymer chains, resulting in faster diffusion. Nevertheless, sorption is usually a heat-releasing procedure, and the solubility of the permeant have a tendency to reduce with a rise in temperature. When the increase in diffusivity is adequate to offset the decline in the permeant solubility which is frequently detected in the case of hydrophilic membranes, the permeability enhances with temperature. The studied temperature dependency of the permeation flux indicates the collective impacts of temperature on the driving force and the permeability in the membrane.

### 5.2. Membrane Swelling and Preferential Sorption

**Figure 13:**
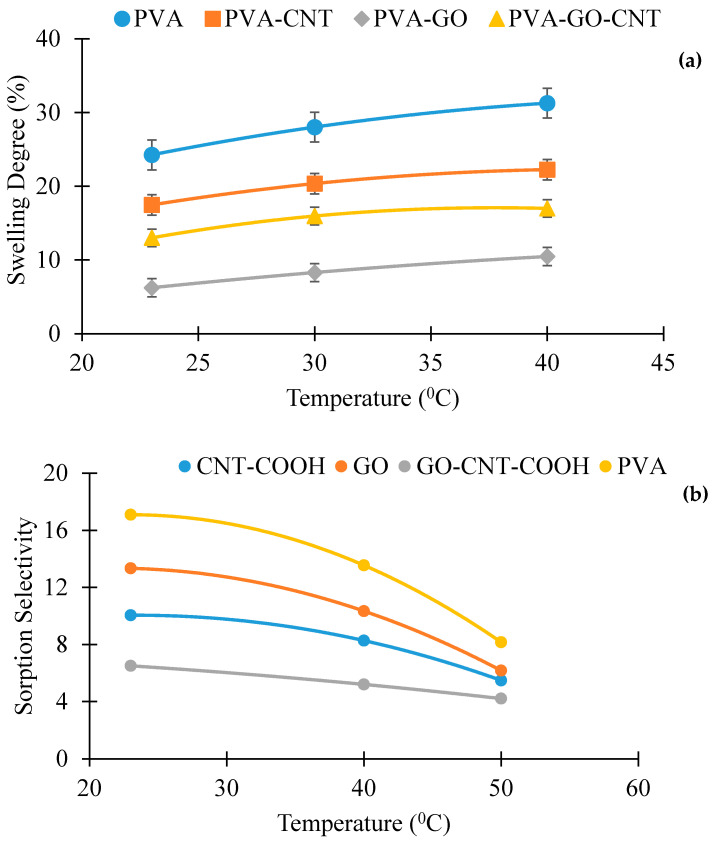
Swelling degree as a function of temperature with 15% water (85% ethanol) in the feed mixture and its corresponding sorption selectivity.

To explore the favored sorption of water and ethanol in the membranes, the quantity and composition of the sorbate in the membrane were established. The uptake of membranes was carried out with 15/85 wt.% water-ethanol solution. The extent of swelling of the membranes in ethanol/water solutions with variation in temperature is presented in Figure 13. With increase in temperature, the swelling degree increases for both pristine and fabricated membranes. By comparison of the swelling information for membranes PVA, PVA-GO, PVA-CNT-COOH and PVA-GO-CNT-COOH, it was observed that the pristine PVA membrane has the highest degree of swelling than the fabricated membranes, which could be due to the presence of higher amounts of hydroxyl groups in the unmodified PVA membrane. The swelling degree of the PVA-CNT-COOH membrane was slightly higher than the PVA-GO membrane and the PVA-GO-CNT-COOH membrane. This suggests that the swelling of the PVA component is limited by the occurrence of hydrophilic functional groups, and the sorption uptakes in PVA and nanomaterial constituents are not independent. Essentially, the reduction in uptake is linked to the robust GO-polymer interactions which, further reduces the availability of hydrophilic groups, may perhaps inhibit the flexibility of PVA chains and significantly reduce the void volume of the cross-linked PVA. To add-on to the increase in crystallinity of PVA/CNT-COOH membrane, there was also a greater interfacial force, i.e., hydrogen bonding, between COOH and OH, causing inhibition of polymer chain mobility which also added to inhibiting membrane swelling in water.

In fact, the impacts of PVA and nanomaterials in the crosslinked blend system to sorption are not linear owing penetrant and the membrane constituents’ interactions. The extent of swelling is indicative of the total uptake of the sorbate in the membrane and cannot be used to determine if and how the sorption of a specific element in the penetrant is affected by a subsequent penetrant element. For determining the preferential sorption which depends very much on the strong interactions of the penetrant–membrane system, the solubility of water and ethanol in the membranes were studied. The sorption isotherms of water and ethanol in the pristine PVA and fabricated membranes at different liquid water concentrations are illustrated in Figure 13. As expected, the membrane hydrophilic nature due to the presence of GO and PVA enables high sorption capacity of water. The water molecules dissolved in the membrane swell the membrane, resulting in free volume generation and chain mobility of the PVA. Ethanol uptake is significantly impacted by water. Pure ethanol can sparingly be dissolved in the membrane, but in the presence of water-swollen membrane, the dissolution of ethanol in the membrane is very rapid. Water amounts increase in the liquid phase results in increasingly swollen membranes and the ethanol uptake improves even with decreasing ethanol concentration in the feed mixture. This continues until ethanol amount in the feed reduces significantly, and subsequently the ethanol solubility starts to decrease with an additional increase in water concentration in the feed. Comparable sorption pattern was seen as well for other membranes studied here. Hauser et al. [88] determined the sorption uptake of water and organic components in binary water-organic mixtures in a crosslinked PVA, and comparable findings on the coupling sorption were found. PVA crosslinking with GA results in dense and compact structures due to contraction of the polymer chains, thus lowering the flux with high sorption selectivity. Yet, in the blend membrane, the addition of the nanocarbons improves the hydrophilicity and mass transfer coefficients thus increasing flux.

### 5.3. Model Calculation of the Theoretical Flux and Diffusion Coefficient

In a binary system like ethanol-water mixture, the pervaporative flux for the ‘*i*th’ and ‘*j*th’ component (*J_i_* or *J*_*j*_; where ‘*i*’ and ‘*j*’ represent ‘*water*’ and ‘*ethanol*’, respectively) through a nonporous dense membrane can be expressed by Fick’s first law [89], as

(5) $$J_{i}=-\left(\frac{\rho_{m}D_{i}}{1-W_{im}}\right)\frac{dW_{im}}{dl}$$

where, *ρ_m_* is the membrane density, *D* is the diffusion coefficient, *W_im_* is the membrane phase water mass fraction and *l* is the membrane thickness.

Considering a very small amount membrane phase water mass fraction, Equation (3) can be reduced to,

(6) $$J_{i}=-\rho_{m}D_{i}\frac{dW_{im}}{dl}$$

The diffusion coefficient of the permeating species depends on the concentration in the membrane and their mutual coupling effect and can be described as [90]:

(7) $$D_{i}=D_{i0}exp\left(W_{im}+\beta W_{jm}\right)$$

(8) $$D_{j}=D_{j0}exp\left(W_{jm}+\gamma W_{im}\right)$$

where, *β* and *γ* are the coupling parameter of ethanol and water, respectively.

Substituting Equation (3) into Equation (2),

(9) $$J_{i}=-\rho_{m}D_{i0}exp\left(W_{im}+\beta W_{jm}\right)\frac{dW_{im}}{dl}$$

The total water flux (here, it is the *i*th component) can be obtained by integrating Equation (9) over the membrane thickness as.

(10) $$\int_{0}^{l}J_{i}dl=-\rho_{m}D_{i0}\int_{W_{imf}}^{imp}exp\left(W_{im}+\beta W_{jm}\right)dW_{im}$$

Since, the permeating components desorbs quickly from the membrane surface at the downstream side, the concentration of the diffusing species at the permeate side can be considered very low and Equation (8) reduces to,

(11) $$J_{i}=\frac{D_{i0}\rho_{m}}{l}\left[exp\left(W_{im}+\beta W_{jm}\right)-1\right]$$

Similarly, for ethanol (*j*th component),

(12) $$J_{j}=\frac{D_{j0}\rho_{m}}{l}\left[exp\left(W_{jm}+\gamma W_{im}\right)-1\right]$$

The diffusion coefficient of the permeating species can be calculated using a linear regression of the permeation and sorption data and comparing the regressed equation with Equations (11) and (12). As both permeating species are highly polar, a plasticization effect has been expected to occur. The values of the plasticization coefficient, *β* and *γ*, were predicted to maintain similarities between the experimental flux and the predicted flux.

Table 3 demonstrates the diffusion coefficient values of water and ethanol at infinite dilution for all membranes along with their corresponding β and γ values. The D_0,water_ values are observed to be much higher than D_0,ethanol_, as the molar volume and kinetic diameter of water is much smaller than ethanol [91]. It can be seen from Table 3 that the diffusion coefficient of water was increased with the incorporation of nanomaterials into the polymer matrix. This may be due to the to the ability of the nanomaterials to influence water–polymer interactions [35,92]. The enhanced diffusion coefficient for PVA-GO-CNT-COOH membranes is mainly due to the mutual interactions of water molecules with GO and fast movement along frictionless CNTs, considered as the ‘fast lanes’ for water flow [93].

## 6. Proposed Mechanism

**Figure 14:**
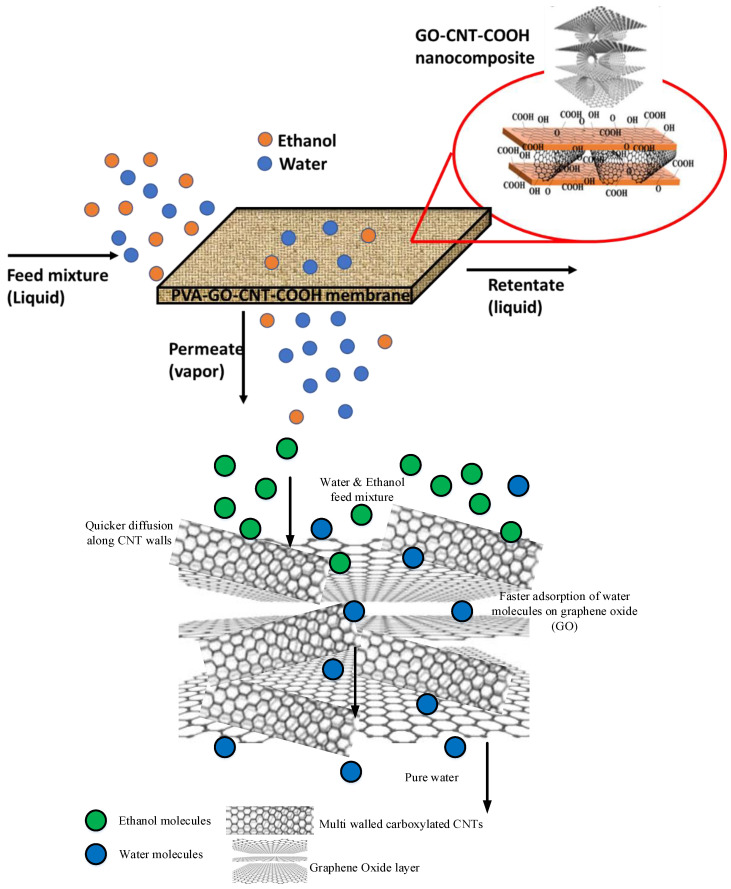
Schematic of proposed mechanism.

The schematic of the proposed mechanism is demonstrated in Figure 14. It is well known that CNTs provide rapid mass transport due to smooth nanotube walls, making CNTs an ideal candidate filler material for membranes [35]. Recent studies have shown that the incorporation of nanocarbons reduce the fractional free volume in membranes [94], and the same has been shown by incorporation graphite into the PVA matrix where solute diffusion was enhanced by this [95]. The GO-CNT incorporation represented significant enhancement in performance over either pure CNTs or GO. The GO-CNT tuned the packing of hydrophilic chains, which in turn decreased free volume and thus improved selectivity. Additionally, it can be inferred that the GO-CNT hybrid provided internal nanochannels which acted as nano corridors for water permeation. These paved ways for enhanced performance in terms of flux and separation factor in dehydrating ethanol from water along with strong interfacial interactions between charged CNT-GO and PVA matrix. In the GO-CNT matrix, the addition of GO to the PVA membrane matrix plays a role in enhancing the partitioning of the water in the membrane because it is significantly hydrophilic [96]. On the other hand, the CNTs are known to provide rapid mass transfer for the water molecules through a membrane [97]. The combination of GO and CNTs is an ideal filler material as it provides both high partition coefficient and rapid mass transfer because we can exploit the synergistic effects of these two nanocarbons.

## 7. Conclusions

PVA/GO-CNT nanocomposite membranes were prepared by adding GO and CNT-COOH into PVA matrix via a casting method using glutaraldehyde as the crosslinking agent and HCl as the catalyst. The 1D CNT-COOH and 2D GO nanosheet established a unified 3D network via π-π interaction in PVA matrix. This 3D network structure, combined with strong interfacial interactions between charged CNT-GO and PVA matrix results in improved separation performance of the fabricated membranes in dehydrating water-ethanol mixtures. The water flux and separation factor of PVA/GO-CNT-COOH membrane (1:1, *w*/*w* of GO-CNT) were 1.43 kg/m^2^.h and 459.04, respectively, at 40 °C and 10 wt.% water in feed, surpassing those of PVA membranes incorporating either GO or CNT-COOH alone. These outcomes suggest that a synergistic effect was accomplished through the combination of GO and CNT-COOH. We believe that the ultrathin GO layer plays a role in molecular sieving resulting in selective permeation. The overall length of diffusion is minimal because of the ultrathin GO layer, permitting rapid permeation. On the other hand, the CNT-COOH confirms high permeation rates with short diffusion lengths along with structural integrity. From an industrial viewpoint, the novel PVA-GO-CNT-COOH mixed-matrix membrane could be a favorable candidate for biofuels production and organic solvent recovery.

## Figures and Tables

**Table 1:** Comparison of dehydration of ethanol through various membranes.

Membrane System	Conditions	Flux (g/m^2^.hr)	Separation Factor	Reference
PDMS-b-PPO Membranes Modified with Graphene Oxide	4.4–70 wt.% water in water-ethanol mixture at 22 °C.	80–90	72–34	[59]
PVA/PAN-Aluminosilicate membrane	Ethanol-water (10 wt.%) at 35 °C	51	141	[60]
PVA–SiO_2_/poly(AN-co-MA) membrane	Ethanol-water (10 wt.%) at 34 °C	44	72.8	[61]
PVA-SiO_2_/PAN membrane	Ethanol-water (10 wt.%) at 60 °C	390	20	[32]
POSS-GO/PDMS membrane	Ethanol-water (5 wt.%) at RT	1347	11.2	[62]
CPVA-g-C3N4-4/PAN membrane	Ethanol-water (10 wt.%) at RT	6332	30.7	[63]
NR/PMMA-RAFT-SiO_2_ hybrid membrane	Ethanol-water (20 vol%) at RT	2244	19	[64]
AgNPs-PVA membrane	Ethanol-water (10 wt.%) at 40 °C	127	43.6	[65]
rGO/PVA membrane	Ethanol-water (20 wt.%) at 50 °C	56	51.2	[66]
PVA-GO-CNT-COOH hybrid membrane	Ethanol-water (10 wt.%) at 23 °C	870	523	This work

**Table 2:** Summary of Contact Angles for Pristine PVA and fabricated membranes.

Membrane	Pristine PVA	PVA-GO	PVA-CNT-COOH	PVA-GO-CNT-COOH
Contact angle (°)	69	65	75	71

**Table 3:** Diffusion coefficients of Water (D_0,water_) and ethanol (D_0,ethanol_) through all the membranes at infinite dilution at 23 °C.

Membrane	D_0,water_ (m^2^/s)	*γ*	D_0,ethanol_ (m^2^/s)	*β*
PVA	3.16 × 10^−9^	0.17	7.91 × 10^−11^	0.0001
PVA-CNT-COOH	2.06 × 10^−8^	0.38	2.06 × 10^−10^	0.00006
PVA-GO	1.58 × 10^−8^	0.4	3.95 × 10^−10^	0.0001
PVA-GO-CNT-COOH	2.37 × 10^−8^	0.31	3.16 × 10^−10^	0.0001

## Data Availability

Not applicable.
